# Traceable Nanoscale Measurements of High Dielectric Constant by Scanning Microwave Microscopy

**DOI:** 10.3390/nano11113104

**Published:** 2021-11-17

**Authors:** Damien Richert, José Morán-Meza, Khaled Kaja, Alexandra Delvallée, Djamel Allal, Brice Gautier, François Piquemal

**Affiliations:** 1 Laboratoire National de Métrologie et d’Essais (LNE), 78197 Trappes, France; damien.richert@lne.fr (D.R.); jose.moran@lne.fr (J.M.-M.); khaled.kaja@lne.fr (K.K.); alexandra.delvallee@lne.fr (A.D.); djamel.allal@lne.fr (D.A.); 2 Institut National des Sciences Appliquées de Lyon, 69100 Villeurbanne, France; brice.gautier@insa-lyon.fr; 3 Institut des Nanotechnologies de Lyon, 69100 Villeurbanne, France

**Keywords:** dielectric constant, high-κ dielectric, least square adjustment method, micrometer-sized capacitor, nanoscale capacitance measurements, PMN-PT, PZT, scanning microwave microscopy, uncertainty

## Abstract

The importance of high dielectric constant materials in the development of high frequency nano-electronic devices is undeniable. Their polarization properties are directly dependent on the value of their relative permittivity. We report here on the nanoscale metrological quantification of the dielectric constants of two high-κ materials, lead zirconate titanate (PZT) and lead magnesium niobate-lead titanate (PMN-PT), in the GHz range using scanning microwave microscopy (SMM). We demonstrate the importance of the capacitance calibration procedure and dimensional measurements on the weight of the combined relative uncertainties. A novel approach is proposed to correct lateral dimension measurements of micro-capacitive structures using the microwave electrical signatures, especially for rough surfaces of high-κ materials. A new analytical expression is also given for the capacitance calculations, taking into account the contribution of fringing electric fields. We determine the dielectric constant values ε_PZT_ = 445 and ε_PMN-PT_ = 641 at the frequency around 3.6 GHz, with combined relative uncertainties of 3.5% and 6.9% for PZT and PMN-PT, respectively. This work provides a general description of the metrological path for a quantified measurement of high dielectric constants with well-controlled low uncertainty levels.

## 1. Introduction

The importance of dielectrics is undeniable in the development of electronic materials and devices [1]. They have contributed for decades in the scaling of metal oxide semiconductor (MOS) and complementary MOS (CMOS) technologies associated with ever-smaller device dimensions down to the sub-10 nm level [2,3], and currently, in three-dimensional (3D) multi-stack and heterogeneous integration technologies [4]. Current applications of dielectrics include interconnect technologies based on materials with low dielectric constant values ε_r_, also noted κ in the field of integrated circuits and semiconductors (κ < 3.9) [1], communication, energy storage, memory, and micro-electro-mechanical systems (MEMS) technologies, which involve high-κ materials (κ > 3.9) [5,6,7,8] and nanoscale capacitors [9,10,11,12,13]. Nevertheless, actual nanostructures and devices of interest remain in most cases far from ideal, showing important local defects and non-homogeneities. This makes their characterization relatively complex. A thorough investigation of parasitic contributions to the actual values becomes thus fundamental for metrological assessments and traceable results to be ensured.

Ferroelectric materials have also gained a rising interest for their applications in micro- and nano-scale actuators for the development of MEMS devices [14]. Thin piezoelectric films, in particular, have been shown to exhibit interesting properties for their use in high frequency and microwave applications [15,16]. 

The knowledge on dielectric constant (the real part of the relative permittivity as well as the dielectric losses) is essential to design thin film or nanopillar capacitors and depends on the frequency range of the targeted applications (kHz for energy storage, GHz for CMOS). Reliable and reproducible information on the relative permittivity as well as other electrical and physical quantities strongly require new metrological tools suitable for nanoscale measurements [2,4,17,18]. This includes development of new reference materials, robust measurement methods and calibrated instruments.

Few works have been reported in the literature on the nanoscale capacitance and dielectric constant measurements of high-κ thin film materials [19]. Scanning probe microscopy (SPM)-based methods for a local electrical and electromagnetic characterization of these materials remain however at a paramount position. Scanning microwave microscopy (SMM) constitutes the most suited method to measure the admittance of dielectric thin films at the nanoscale [20,21,22,23].

In this work, we present a detailed study of high-κ based multi-layered structures developed for the local measurement of high dielectric constants, namely in the range of 400–700, using SMM. The studied structures are based on two materials, PbZrTiO_3_ (Lead Zirconate Titanate) and PbMN-PbTiO_3_ (lead magnesium niobate-lead titanate), thereafter referred to PZT and PMN-PT. These dielectrics are gaining ever-growing importance for microwave applications in the development of tuneable devices at microwave frequencies such as delay lines, filters and phase shifters [8,13,15,24,25]. They are also involved in high frequency switches [13,26] and magnetic sensors for medical needs [27,28]. 

Our structures exhibit, however, several structural defects related to the rough and non-homogeneous nature of the high-κ layers as well as to local interlayers detachments and gaps. This leads to considerable variations of the measured capacitances and dielectric constants dependent on the size of the capacitive structures, material density and the frequency of the incident microwave. 

The present paper describes a detailed metrological approach to investigate non-ideal, yet real-life, nanostructures and extract the dielectric constant value of high-κ materials.

## 2. Materials and Methods 

### 2.1. Experimental Set-Up

Experiments are performed using a 5600LS AFM (Commercial instruments are identified in this paper for technical clarity and do not imply recommendation or endorsement by the authors) interfaced with a N5230C Vector Network Analyser (VNA) from Keysight Technologies, Santa Rosa, CA, USA. Bulk platinum conductive AFM probes (Rocky Mountain Nanotechnologies, Salt Lake City, UT, USA) are used (spring constant *k*_S_ = 18 N·m^−1^) in contact mode. The AFM tip is connected to the RF source/receiver of the VNA through a homemade Mach-Zehnder type microwave interferometer, enabling the system to operate at numerous frequencies in the range between 0.5 GHz and 6.0 GHz [29]. The AFM is placed on an active anti-vibration table inside a glove box workstation (Mbraun, Garching, Germany) under nitrogen gas atmosphere at room temperature and small relative humidity (*RH* < 1%). The whole set-up is installed in a shielded room, fitted with a controlled air conditioning system [29]. In addition, a calibrated AFM [30] (Veeco, Plainview, NY, USA) and a scanning electron microscope (SEM, Zeiss Ultra Plus equipped with an In-Lens detector and charge compensation) [31] are used to traceably measure dimensional parameters of the samples under study.

### 2.2. Samples

#### 2.2.1. Reference Samples

Two capacitance calibration kits (MC2 Technologies, Villeneuve-d‘Ascq, France), labelled A61 and A64, served as reference samples for the SMM calibration procedure. The calibration samples are composed of identical patterns, each including 48 micro-capacitors formed by a SiO_2_ dielectric layer (with different thicknesses by plateaus) sandwiched between circular gold pads and a highly boron-doped p-type Si (100) substrate. These metal oxide semiconductor (MOS) capacitors are designed for capacitance values ranging from 200 aF to 10 fF [21]. We have recently reported on a comprehensive metrology characterization of these calibration samples [32], demonstrating a combined uncertainty of 3% at one standard deviation (*k* = 1) for capacitance measurements at the operating frequency of 3.6 GHz.

#### 2.2.2. Piezoelectric Samples

**Figure 1:**
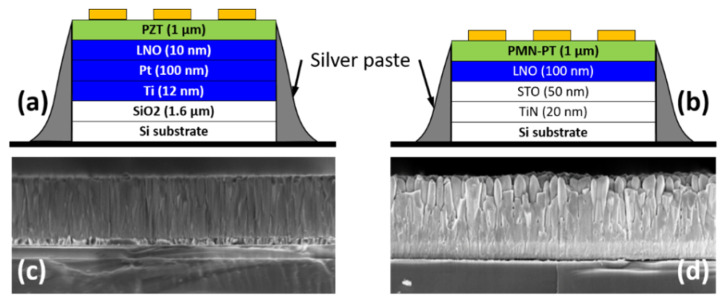
(**a**,**b**) Schematic representations of the multilayer structure of the PZT and PMN-PT samples, respectively. (**c**,**d**) Correspond to cross-section SEM secondary electron images using the In-Lens detector.

Two high-κ samples are investigated in this work, hereafter called PZT and PMN-PT, provided by Electrosciences Ltd (Farnham, UK). Each sample is composed of multiple layers deposited on a silicon substrate, as shown in Figure 1. The PZT sample (Figure 1a) is formed by 1 µm thick layer of lead zirconate titanate on top of a multilayer stack (10 nm thick LaNiO_3_, 100 nm thick Pt and 12 nm Ti layers), and a 1 μm thick SiO_2_ layer on the Si substrate. The PMN-PT sample (Figure 1b) is formed by a 1 µm thick film of lead magnesium niobate lead titanate on top of different materials (100 nm thick LaNiO_3_, 50 nm thick SrTiO_3_, and 20 nm thick TiN) on the Si substrate. SEM images (Figure 1c,d) show the cross sections of the two samples PZT and PMN-PT, respectively. Microcapacitor structures are fabricated (Renatech clean rooms) by the deposition of 50 nm thick circular gold pads on top of the high-κ layers. A set of 225 patterns are made on the samples; each pattern is formed by 15 circular pads with diameters varying between 100 nm and 2.2 µm. Side electrical contacts have been created using silver paste to shortcut the insulating layers under the high-κ films and connect them to ground, as shown in Figure 1. The added contact alters the original micro-capacitor structures to include only the high-κ dielectric layer sandwiched between the top gold pad electrode and the grounded back electrode. 

### 2.3. Calibration Method

SMM experiments consist in measuring the sample’s impedance through a measurement of the reflection coefficient given by the ratio of the reflected to the incident microwave signals. Nevertheless, the use of an impedance matching circuit between the AFM probe and the vector network analyser induces three experimental error terms (*e*_00_, *e*_01_, *e*_11_). This creates a deviation between the actual and the measured reflection coefficients. A calibration procedure using reference samples (here A61, A64 structures from MC2 technologies) is required to correct for the induced experimental errors. A modified short open load (mSOL) calibration method is used to this end [33]. The method applies the classical one-port VNA calibration using three known capacitance standards (triplet) to determine the three experimental error terms. These standards are established from reference capacitor triplet selected on the MC2 samples A61 or A64 [32]. The triplet capacitance standards are chosen on the thickest SiO_2_ layer (4th plateau) of one reference sample. This reduces significantly the combined uncertainty level to 3% (*k* = 1) resulting from the depletion capacitance at the SiO_2_/Si interface as well as the observed parasitic series capacitances [32]. The error terms are found through a comparison between the measured SMM values and a thorough numerical modelling of the standard micro-capacitor structures (see Section 3.1). The actual reflection coefficient *S*_11_ is related to the measured *S*_11,m_ by:

(1) $$S_{11}=\frac{S_{11,m}-e_{00}}{e_{01}+e_{11}\left(S_{11,m}-e_{00}\right)}.$$

The impedance of the sample follows as:

(2) $$Z_{s}=Z_{0}\frac{1+S_{11}}{1-S_{11}},$$

with *Z*_0_ = 50 Ω is a reference impedance. The measured capacitance of the sample is determined at the selected frequency of the microwave measurement.

### 2.4. Measurement Protocol

In SMM, maps of *S*_11,m_ are recorded by scanning the conductive AFM tip in contact with the sample across a given area. The *S*_11,m_ images are processed using a differential approach in which Δ*S*_11,m_ = *S*_11,m_ − *S*’_11,m_ is determined for each scanning line. Δ*S*_11,m_ corresponds to the difference between the raw *S*_11,m_ signals measured on individual capacitors *C*_i_ and the *S*’_11,m_ signals measured on the dielectric layer surrounding the capacitor_._ This approach is intended to exclude the background capacitive signal, thus reducing stray capacitances involved in the measurement [32]. Moreover, the sample configuration is designed such that the investigated samples are placed in the immediate vicinity of the reference sample as previously reported in [29,32]. This is specifically intended to avoid large variations in the local electromagnetic environment of measured samples. 

**Figure 2:**
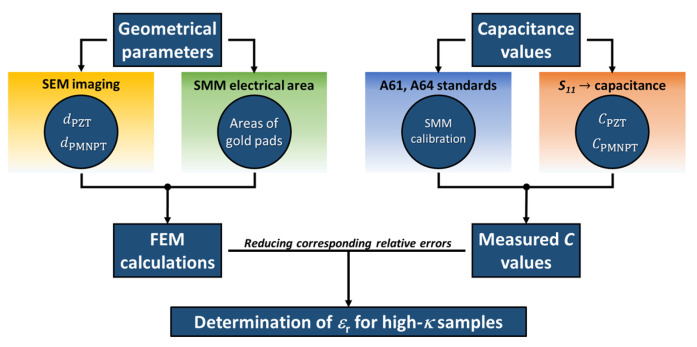
Schematic diagram describing the workflow steps of our measurement and simulation protocol for the determination of the dielectric constant of the high-κ samples. *d* and *C* denote the measured thickness and capacitance of the PZT and PMN-PT dielectric layers, respectively.

Calibration measurements are initially conducted on the standard SiO_2_ micro-capacitors samples (A61 or A64) and the error terms are determined. The actual impedance is then extracted for the PZT and PMN-PT samples using the same error terms. Nevertheless, the determination of the relative dielectric constant of the investigated samples requires an additional modelling of their capacitive structures. This implies a traceable metrological characterization of their structural dimensions (layer thickness and gold electrode area) to be used for the capacitances’ calculations. The workflow of our protocol is schematically depicted in Figure 2. In this work, we describe the different methods adopted to this end and we propose novel approaches to overcome intrinsic difficulties related to complex structures and considerably rough surfaces.

## 3. Results

### 3.1. Capacitance Model 

#### 3.1.1. Theory

In a first approximation, the capacitance of the micro-capacitors on the SiO_2_ standards and the investigated high-κ samples are estimated using the well-known parallel-plate capacitance *C*_P_ of the disk capacitor calculated from the uniform field model:

(3) $$C_{p}=\varepsilon_{r\;}\varepsilon_{0\;}\frac{A}{d},$$

with ε_r_ as the relative permittivity of the dielectric layer, ε_0_ as the vacuum dielectric constant, *A* as the area of the top electrode, and *d* as the thickness of the dielectric layer. However, this relation only holds for the cases where the electric field between electrodes can be considered as uniform. This is mostly valid when the area of the electrodes considerably exceeds the thickness of the dielectric layer. When the electrode’s area becomes comparable (or smaller) to the dielectric thickness, the effect of fringing fields, originating from side effects in the capacitive structure, gain an important weight and contributes to a large part of the measured values [17]. Additional effects (1% correction) must also be taken into account in the case of our standard capacitive (SiO_2_) structures. They are namely related to depletion capacitances at the SiO_2_/Si interface and to surrounding stray capacitances [32]. To this end, we apply finite element modelling methods (FEM) to calculate capacitances *C*_FEM_ using COMSOL-Multiphysics with the AC/DC module. The FEM calculations rely in particular on the measured values of micro-capacitive structures’ geometrical parameters, such as the equivalent radius *R* (related to the area) and the height *h*_pad_ of the gold pad and the thickness *d* of the dielectric layer. 

For the capacitance standards based on SiO_2_, the traceable geometrical parameters have been determined following our recent work in [32]. The micro-size capacitive structures in this case present a small dielectric thickness compared to the area of the electrodes. The geometrical condition *d* << *R* (for a uniform field) is thus satisfied, which validates the use of Equation (3) in the corresponding analytical calculations. For this, we considered ε_r,SiO2_ with a relative uncertainty of 1%. Nevertheless, even if the effect of the fringing fields is small for the case of standard samples’ structures, we still consider it as a minor additional correction term to the first approximation expression in Equation (3). An analytical expression of this correction has been found empirically and leads to an error term lower than 20% for *R*/*d* > 10 in a good agreement with the numerical calculation at the level of 1% [32]. 

For the case of the high-κ samples studied here, the dimensions of the circular gold electrodes and dielectric layers’ thicknesses are described in detail in Section 3.1.2 with *R*/*d* ≤ 1, which makes the contribution of the fringing fields to the measured capacitances high. It is therefore mandatory to consider a new analytical expression to correct the first approximation (uniform field) of parallel-plate capacitor *C*_P_. For this, we found the following expression:

(4) $$C=C_{P\;}\left[1+\left(1+\frac{1}{3ln\left(\varepsilon_{r}\right)}\right)h\left(d,R\right)\right],$$

where

(5) $$h\left(d,R\right)=\left[1+\gamma^{\prime }ln\left(\frac{d}{R}\right)\right]\frac{d}{R}\;,$$

and γ′ is an adjustable parameter depending slightly on *h*_pad_, γ′ = 0.097 for *h*_pad_ = 50 nm.

**Figure 3:**
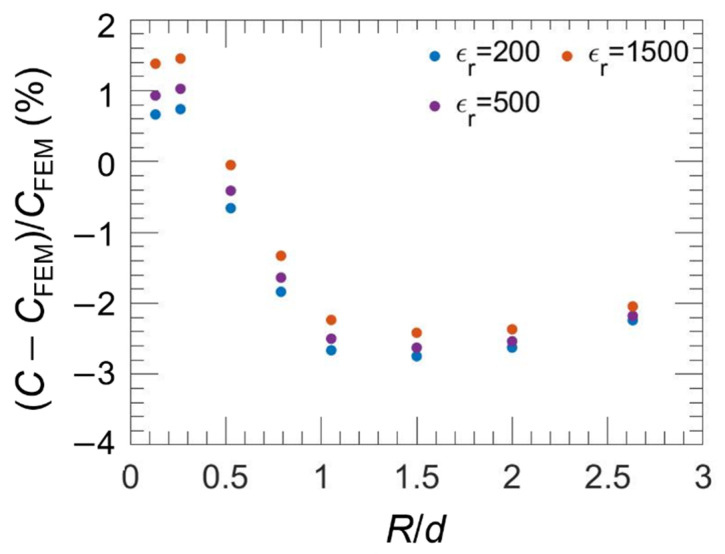
Relative differences (*C*—*C*_FEM_)/*C*_FEM_ in% as a function of *R*/*d*, with *R* ranging from 0.10 µm to 1.2 µm and *d* fixed at 1 µm, for four values of ε_r_ (200, 500, 1500).

For *d*/*R* ranging from 2 to 10, *h*(*d*,*R*) increases almost linearly as a function of *d*/*R* with a slope weakly dependent on ε_r_ in agreement with [34]. In case of *d*/*R* >> 1, this leads to a first order approximation

(6) $$C=\varepsilon_{r}\varepsilon_{0}\pi R,$$

independent of the electrode separation as expected for capacitance of uncoupled circular electrodes [35,36]. The capacitance calculation using the relations (3) to (5) agrees with FEM calculation at the level of 3% for 0.2 < *d*/*R* < 2.6 and for a wide range of ε_r_ values, from 200 to 1500, as shown in Figure 3. Moreover, the observed deviations weakly depend on the ε_r_ values, without exceeding 1%. Therefore, the FEM approach will be preferred to analytical ones for a precise capacitance calculation on high-κ samples. However, the analytical method will be applied to evaluate the uncertainty of the capacitance calculation (by propagating the uncertainties on input values *d* and *R*) and then to estimate the uncertainty on the dielectric constant determination. The uncertainty on the correction to apply for analytical calculated values is estimated less than 1% corresponding to the dependence on ε_r_. Moreover, the analytical approach (simpler and less time consuming than FEM) can be helpful to evaluate influence of parameters such as the frequency.

#### 3.1.2. Dimensional Measurements

In this section, we detail our approach for the determination of the dimensional parameters of the high-κ samples, i.e., the thickness of the dielectric layers and the gold pads’ areas.

- Layer thickness

Cross sections of the PZT and the PMN-PT samples are imaged with scanning electron microscopy (Zeiss Ultra Plus) with secondary electrons mode as shown in Figure 1c,d, respectively. We measure the layers’ thickness *d*_PZT_ = (950 ± 10) nm for the PZT layer and *d*_PMN-PT_ = (964 ± 26) nm for the PMN-PT layer. The uncertainties are obtained from the analysis of multiple averaged line profiles. However, cross-sectional SEM images show important structural differences between the two samples. While both dielectric layers seem to have pillar-like structures, the PMN-PT film shows a strong structural roughness which is expected to impact the quality of the sample’s surface.

- Gold pad areas

**Figure 4:**
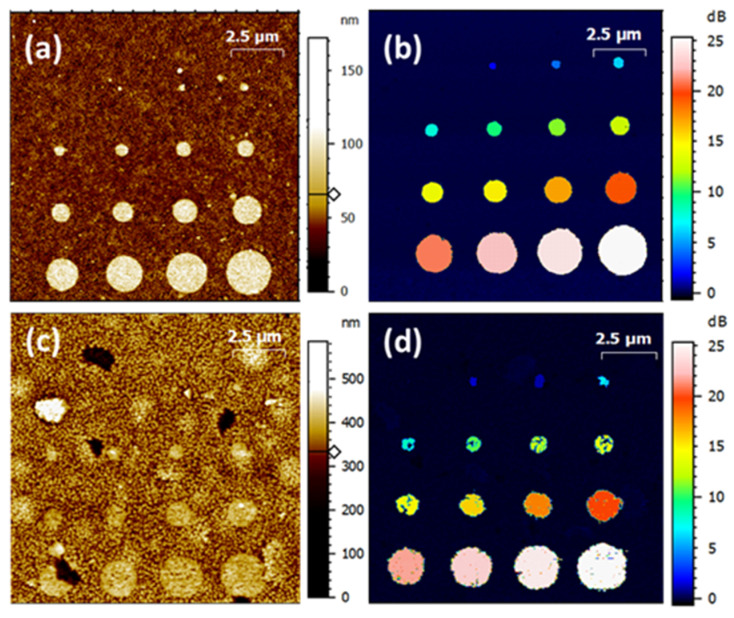
Tapping mode AFM images of (**a**) PZT and (**c**) PMN-PT samples taken with the LNE’s calibrated AFM. Δ*S*_11,m_ magnitude maps of (**b**) PZT and (**d**) PMN-PT samples obtained with Keysight’s SMM.

We use our calibrated AFM [30] to measure the surface topography of the gold pad structures on the PZT and PMN-PT samples. For this, a low scanning rate (7 µm/s) and high pixel-resolution images (2048 pixels square) are applied. Figure 4a,c shows the metrological AFM topography obtained on PZT and PMN-PT samples, respectively. In correlation with the cross-sectional SEM images in Figure 1c,d, the PMN-PT sample presents a higher surface roughness compared to the PZT sample. We measure the surface roughness using the root mean square height parameter (*S*_q_) for both samples at *S*_q_ = 13.4 nm for PZT and *S*_q_ = 37.4 nm for PMN-PT. The thickness of the circular gold pads deposited on both surfaces is 50 nm. 

Finding the gold pad areas on the PZT sample is straightforward using surface analysis techniques (including background adjustment, features masking and particles’ threshold analysis). However, the high *S*_q_ value compared to the pad’s thickness in the case of the PMN-PT sample makes the determination of the gold pad areas from AFM topography challenging. 

**Figure 5:**
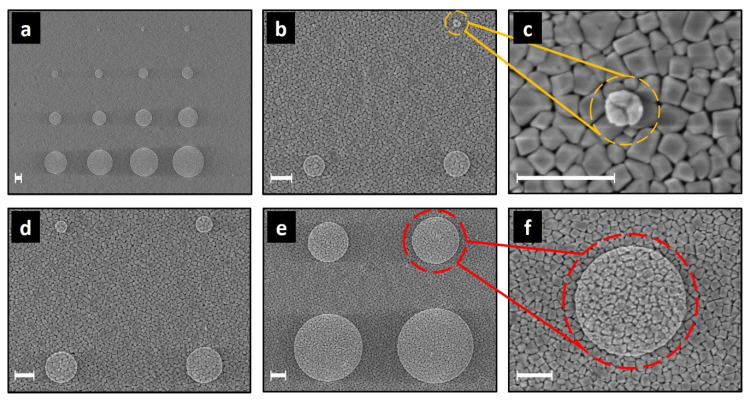
SEM images of a pattern formed by 15 gold pads (**a**). Different zones of this pattern are imaged: Top right in (**b**), middle left in (**d**) and bottom right in (**e**); (**c**,**f**) high resolution images of individual pads. All scale bars correspond to 400 nm.

For this, an alternative approach has been adopted using the so-called “electrical pads’ area”. We make use of the SMM electrical signature of the micro-size capacitive structures, as the *S*_11,m_ signal over the gold pads is considerably higher than the surrounding surface. This is mainly because the capacitive structure on the surface surrounding the gold pads is actually formed between the tip apex and the rest of the dielectric film. The corresponding reflection signal is thus extremely small compared to that originating from the capacitive structures on the gold pads. This results in a well-defined contrast on SMM images (here Δ*S*_11,m_ magnitude) delineating the circular gold pads, as shown in Figure 4b,d. Our approach consists in using the well-defined imaging results on the PZT sample to establish a correlation factor *N* between the areas’ dimensions measured on the Δ*S*_11,m_ maps and the topographical dimensions of the gold pads. *N* is then applied, as a correction factor, to the PMN-PT electrical map (Figure 4d) to back-calculate the corresponding topography dimension of the gold pad areas in this case. Nevertheless, to reduce uncertainties related to the tip convolution in AFM topography measurements, we use SEM imaging of the gold pads on the PZT sample to measure the topographical dimensions, as shown in Figure 5. We note that the correction factor is given by the ratio of the “electrical” to the “topographical” gold pad area measured by SEM (*N* = *A*_elec_/*A*_topo_) for each structure on the PZT sample, as listed in Table 1 below. As a result of this manipulation, we determine the corrected gold pads’ areas for the PMN-PT sample that we further use for the FEM calculations of the micro-structures capacitances.

Table 1 gives the combined uncertainty for each pad area of PZT sample. This uncertainty is calculated from the root sum square (RSS) of two components: (i) the repeatability computed on a set of three SEM images, and (ii) the SEM calibration uncertainty. 

### 3.2. Capacitance Measurements

**Figure 6:**
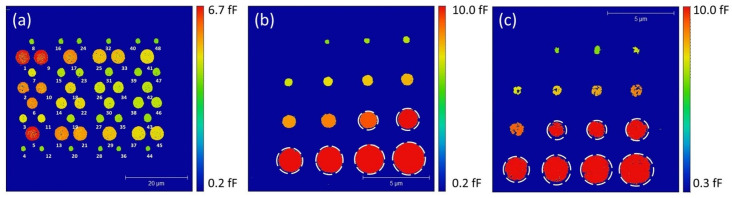
Capacitance maps of the samples at VNA frequency of 3.67 GHz. The circled pads are out of the calibration range. (**a**): A64; (**b**): PZT; (**c**): PMN-PT.

As described in the Section 2, the capacitance maps of the high-κ samples are determined from the measured values of Δ*S*_11,m_ corrected using the SMM calibration protocol, at a given microwave frequency (here *f* = 3.67 GHz). The mSOL method is applied using the A61 and A64 reference samples as described in details in [32]. Figure 6a shows the capacitance map measured on the reference sample A64 with the numbering of the different capacitive micro-structures. A set of three capacitances (triplet) is selected from these values to extract the three correction factors (*e*_00_, *e*_01_, *e*_11_) for the SMM calibration. We used a triplet of capacitances in a way to cover all the range of possible values associated with the A64 sample (from 0.3 fF to 10 fF). The SMM is thus calibrated within this range of capacitances. 

The maps of the measured capacitances for the PZT and PMN-PT samples (shown in Figure 6b,c, respectively) are obtained using the corrected values of *S*_11_ and the samples’ impedance following Equations (1) and (2). The capacitances measured on the structures highlighted with dashed circles are outside of the SMM calibration range as offered by the A64 reference sample. 

#### 3.2.1. Dielectric Constant Determination

**Figure 7:**
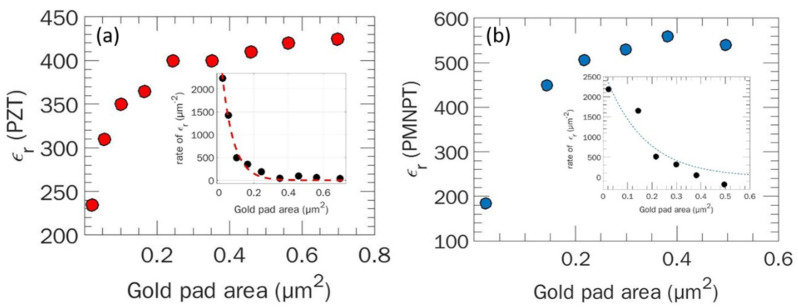
Dielectric constant ε_r_ as a function of gold pad area on PZT (**a**) and PMN-PT (**b**) and its associated derivative with respect to the pads’ area.

Following the workflow in Figure 2, the capacitances of each micro-structure on the PZT and PMN-PT samples are calculated using FEM simulations. Calculations require a prior knowledge of the structures’ geometry (gold pad areas and dielectric thickness), as well as the high-κ dielectric constant. However, being initially unknown, values of the dielectric constant for each sample is input to the calculations and the resulting FEM capacitance, for each micro-structure, is compared to the corresponding measured value. The dielectric constant is thus found by adjusting this input until the difference *C*_FEM_ – *C*_exp_ = 0 (same for the PMN-PT sample). We obtained different values of ε_r_ for each sample depending on the area of the gold pad, as shown in Figure 7.

As shown in the inserts of Figure 7, the rate of change in ε_r_ as a function of the gold pad areas indicates that the values of the relative permittivity tend to stabilize for the larger micro-capacitive structures. These observations cannot be attributed to local changes in the dielectric films since the layers are homogenous from a dielectric point of view. Nevertheless, the structural variations identified from SEM images suggest the contribution of additional parasitic capacitances. These are mainly related to a combination of factors including crystallographic orientation of the PZT grains (Figure 5c,f) and surface roughness resulting in the presence of interfacial voids in the layers that leads to a series capacitances added to those of the dielectric films. Hence, the parasitic capacitance contribution is significantly more important for smaller pads than it is for larger one which explained why the dielectric constant computed for larger pads does not change as much as the one associated with smaller pads.

**Figure 8:**
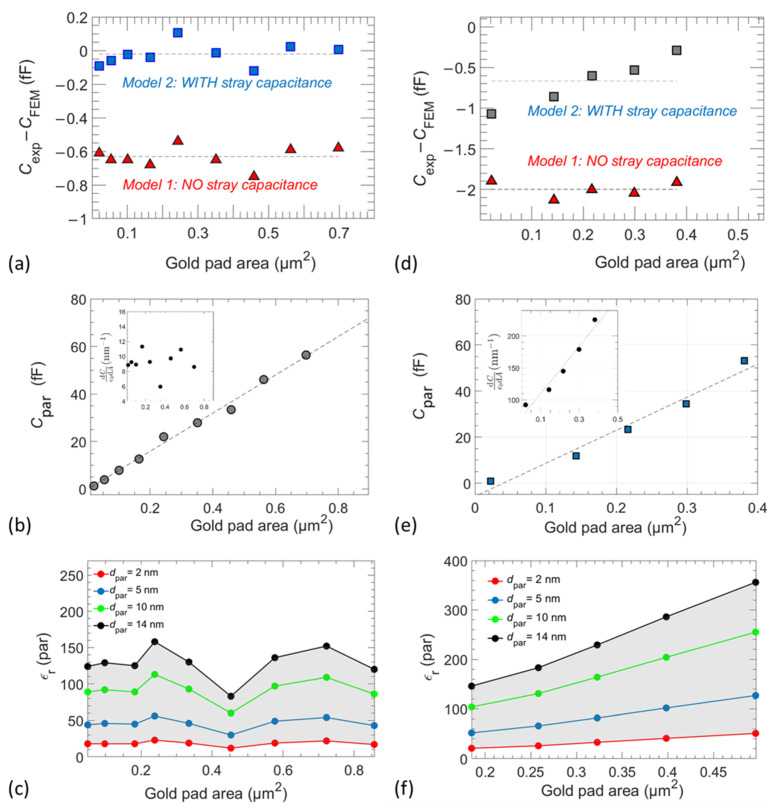
Study on PZT and PMN-PT sample at a frequency of 3.67 GHz; (**a**,**d**): Difference between *C*_exp_ and *C*_FEM_ as a function of the gold pad area on PZT (PMN-PT). (**b**,**e**): Parasitic capacitances with a thickness given the void volume at the PZT (PMN-PT) Surface; (**c**,**f**) corresponding dielectric constants of the parasitic capacitances. Insets in (**b**,**e**) show the derivative of the capacitance with respect to the gold pad area ε_0_^−1^d*C*/d*A*.

As a first step, we model the capacitance for each micro-structure (*C*_FEM_) neglecting the parasitic capacitances contribution (hereafter called model 1). For this, we select a value of ε_r_ close to the range for which the rate is around zero, as in the insets of Figure 7a,b. The value is empirically adjusted until the difference between the experimentally measured and the calculated capacitance for each structure is virtually constant (i.e., *C*_FEM_ – *C*_exp_ = const.), as shown in Figure 8a,d. 

In a second step, we consider the model of a parasitic capacitance *C*_par_ in series with the capacitance due to the high-κ layer (hereafter called model 2). Thus, the measured capacitance for each micro-structure is accounted as:

(7) $$\frac{1}{C_{\exp}}=\frac{1}{C_{\mathrm{high}-k}}+\frac{1}{C_{\mathrm{par}}}=\frac{1}{C_{\mathrm{high}-k}}+\frac{d_{\mathrm{par}}}{\varepsilon_{0}\varepsilon_{\mathrm{par}}A},$$

where *d*_par_ and ε_par_ are the equivalent thickness and dielectric constant of the parasitic capacitance, respectively. This capacitance originates mainly from the surface roughness of the PZT and PMN-PT samples creating local voids at the interface between the deposited gold pads and the samples’ surface. 

**Figure 9:**
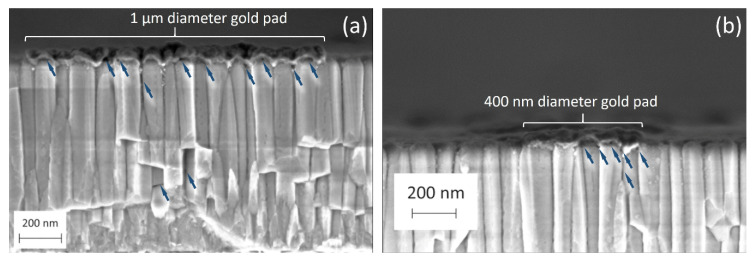
SEM images for cross-section PZT sample taken across (**a**) a 1 µm diameter gold pad and (**b**) a 400 nm diameter gold pad. Blue arrows points to visible voids at the interface as well as among the pillar-like bulk structure of the underlying films.

Figure 9 shows SEM images on a cross section of the PZT sample cut across the gold pads. In addition, energy-dispersive X-ray spectroscopy (EDS) using an Oxford Ultim Extreme detector at 5 kV (not shown here) is conducted on the different regions observed on the SEM images to confirm the nature of the gold pads. Local voids and several imperfections at the gold/PZT interface are clearly noticeable (blue arrows for guidance). In addition, voids across the bulk of the pillar-like structure of the film underlying the gold pads are also observed. The ensemble of these voids creates an equivalent parasitic layer implying the additional *C*_par._ SEM images are obtained across two gold pads of different diameters (1 µm in Figure 9a and 400 nm in Figure 9b). The density distribution of the observed voids is clearly dependent on the size of the gold pads, which explains the dependency of the parasitic capacitance on the area of the pads as observed in Figure 8b. The capacitive behaviour, as measured by SMM, clearly indicates a similar situation for the PMN-PT sample with an even larger influence of the parasitic capacitance due to the higher roughness of this sample. 

Ferroelectric materials such as PZT have been reported to exhibit hydrophilic properties [37,38]. It is therefore expected that confined water could potentially occupy some voids observed at the interface under the gold pads. A picture of the equivalent parasitic capacitance could thus consist of an arbitrary combination of confined water, air-filled voids as well as local peaks of high-κ material forming the surface roughness under each gold pad. 

By considering this model, we adjust the value of the parasitic capacitance until the difference between the measured and FEM-calculated capacitances is set around zero (i.e., *C*_exp_ − *C*_FEM_ ≈ 0*)*, as shown in Figure 8. From measurements carried out on the PZT sample at the frequency *f* = 3.67 GHz, we find a dielectric constant value ε_r,PZT_ = 445 ± 16. In case of the PMN-PT sample, two sets of measurements have been performed, one at 3.67 GHz giving a value ε_r,PMN-PT_ = 650 ± 59 and the other at 3.60 GHz, leading to a value ε_r,PMN-PT_ = 630 ± 67. The weighted mean value of these two values is equal to ε_r,PMN-PT_ = 641 ± 44. 

These values were obtained by using a software [39] to plot the calculated capacitance as a function of the experimental one with the corresponding uncertainties detailed in Section 3.3. Then, by applying a generalized least squares—generalized Gauss Markov regression (GLS-GGMR), the slope was extracted, and the dielectric constant value was adjusted to obtain a unity slope. The uncertainty on the slope gives the uncertainty on the dielectric constant value. The three ε_r_ values calculated from the GLS-GCMR method were successfully validated by performing statistical tests involving χ^2^ and Birge ratio values [40,41,42,43]. The higher uncertainty on the PMN-PT dielectric constant value is due to the larger dimensional measurement’s errors. 

Figure 8b,e shows the variation of the parasitic capacitance as a function of the increasing area of the gold pads for the PZT and PMN-PT samples, respectively. In both cases, the parasitic capacitance varies in the range of 0 fF to 80 fF in a somewhat linear fashion as indicated with the linear fit introduced as a guide for the eye. For the PMN-PT sample, strong uncertainties on the small gold pads due to dimensional measurements give rise to non-consistent values for parasitic capacitances that have been excluded from the linear fit. Nevertheless, the first derivative of the parasitic capacitance with respect to the gold pad areas, as shown in the insets of Figure 8b,e, suggests that the actual variation of *C*_par_ has increasing dependence on the area of the gold pad electrodes. In this regard, the polycrystalline nature of the PZT [16,44] and PMN-PT surfaces in our case is expected to have an important role in a pronounced effect on the dispersion for smaller pads compared to larger pad areas, where an averaging of the polycrystalline effect prevails, as clearly visible on the SEM images in Figure 5c,f.

Using the model of a parasitic capacitance, as described above, an equivalent parasitic layer is introduced to account for the variations of *C*_par._

Equation (8) shows that the ratio of the equivalent permittivity to the equivalent thickness of this parasitic layer is directly proportional to the first derivative of the parasitic capacitance:

(8) $$\frac{1}{\varepsilon_{0}}\frac{dC_{\mathrm{par}}}{dA}=\frac{\varepsilon_{\mathrm{par}}}{d_{\mathrm{par}}}.$$

The quality of the interfacial layer under the gold pads is directly dependent on the surface roughness of the high-κ samples. The measurement of the actual interface roughness under the gold electrodes is not accessible. Nonetheless, the higher the roughness, the higher the equivalent thickness of the parasitic layer would be. For this, we consider a range of different values for the parasitic capacitance thickness *d*_par_ between 2 nm and 14 nm. While the lowest value reflects low local roughness under a gold pad, the highest value of 14 nm corresponds to the average peak-to-valley value calculated over the entire surface excluding the circular gold pads. Using these values, we extract a bounded range for the possible variations of the equivalent relative permittivity of the parasitic capacitance ε_par_ as shown in Figure 8c,f for the PZT and PMN-PT samples, respectively.

We find that, for the PZT sample, the equivalent parasitic permittivity ε_r,par_ remains mostly below 80, except for the higher values of *d*_par_. These values are well below the extracted dielectric constant for the PZT film (i.e., ε_r,PZT_ = 445 ± 16). This suggests that the equivalent parasitic layer is mostly formed by air voids and potentially confined water. Recently reported results on the anomaly low permittivity of confined water, where ε_r_ ≤ 80 [45], go alongside this suggestion. The variations in ε_r,par_ for the PMN-PT sample shows a similar behaviour for the case of a low roughness interface represented by small values of *d*_par_. However, the AFM analysis in Figure 4 clearly indicates a much higher surface roughness for the PMN-PT sample. Thus, the higher values of *d*_par_ (i.e., *d*_par_ = 10 and 14 nm) constitute a better representation of the variations in the dielectric constant of the parasitic capacitance for the case of PMN-PT. It is noticeable in this case that ε_r,par_ is mostly higher than 80 (dielectric constant of bulk water), practically for all gold pad areas, as shown in Figure 8f. ε_r,par_ increases as the area of the gold electrodes increases, reaching values comparable to that extracted for the PMN-PT film (i.e., ε_r,PMN-PT_ = 641 ± 44). This points towards the fact that the rough PMN-PT surface leads to a parasitic layer under the gold pads mostly incorporating peaks of the ferroelectric material with a lower density of voids, especially for larger gold pads. 

This analysis highlights the crucial role of the interfacial surface roughness between the gold top electrodes and the surface of the high-κ sample. As pointed out in a theoretical study in [46] on the effect of one electrode’s roughness on the capacitance of a parallel-plate capacitor, the higher the surface irregularities and roughness of the electrode are, the higher is the capacitance. The interface roughness under the gold pads in the case of our samples increases the effective area of the top electrode for the different micro-capacitor structures. It therefore naturally follows that the larger gold pad areas incorporate a stronger contribution of the parasitic capacitance as observed experimentally in Figure 8b,e.

#### 3.2.2. Frequency Measurements

**Figure 10:**
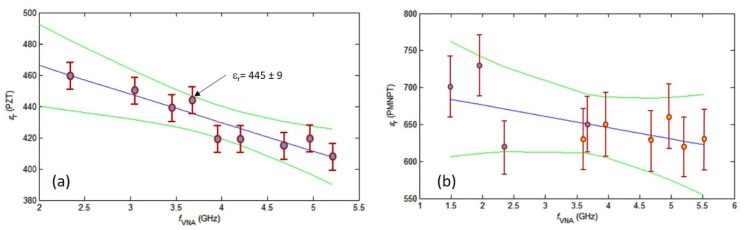
Dielectric constant of PZT (**a**) and PMN-PT (**b**) samples as a function of the VNA frequency. The error bars correspond to the combined uncertainty.

To the best of our knowledge, few results have been reported in the literature regarding the values of the dielectric constant of the high-κ materials (PZT and PMN-PT) in the microwave range [15,47,48,49]. The study of the frequency dependence of this value is of key technological importance as it dictates the response of dielectric materials in high-frequency electronic applications. For this, we perform a series of measurements by varying the microwave frequency in SMM between 1.49 and 5.53 GHz as shown in Figure 10. The upper limit is dictated by the available range of frequency on the VNA used in this work. 

Figure 10a shows the variation of ε_r_ for the PZT sample in the range of frequency stated above. A slight decreasing tendency is noticeable as the microwave frequency increases (blue line). This decrease is in a good agreement with results reported in [47]. It is noteworthy that a weighted least square regression applied to the set of values leads to a slope of Δε_r_/Δ*f* = (18.4 ± 1.6)/GHz. It is much more hazardous to conclude on a decreasing of ε_r_ for the PMN-PT sample as shown in Figure 10b. Moreover, the series of measurements in this case has been performed on two split sets of gold pad structures. This is mainly due to the deterioration of the electrical contrast on the first set of gold pads. The results of the first set correspond to the red markers and the orange markers for the second set. 

### 3.3. Uncertainty Budget

The uncertainties on the resulted values of the high-κ dielectric constants derive from a series of uncertainties related to the various steps in our metrology workflow described thus far (including calibration, characterization and modelling steps). Table 2 details main uncertainty sources corresponding to each sample characterized in the present work. 

The uncertainty on the calculated capacitance values is estimated from the RSS of the uncertainty related to pads’ area, dielectric thickness and analytical expression given by the relations (3) to (5). The same results have been obtained using the FEM method.

The uncertainty of the measured capacitance values is equal to the RSS of the type A uncertainties (combining repeatability and the standard deviation of the histogram Gaussian distribution of each capacitance) and type B uncertainty mainly due to the SMM calibration uncertainty. The other type B uncertainties are related to the residual influence of stray capacitances and relative humidity leading to a combined uncertainty with a conservative value less than 0.8% [32]. The uncertainties corresponding to errors due to stray capacitances have been estimated using the analytical expressions given in [50,51,52]. The sum of residual errors due to the stray capacitances occurring between the sample (metallic pads and dielectric top surface), on one side, and the cantilever, the tip cone and the tip apex, on the other side, does not exceed 18 aF. Considering a rectangular distribution and the measured capacitance range, the corresponding relative uncertainty varies between 0.04% and 1.8%.

## 4. Discussion

We demonstrate that SMM is a powerful tool to quantify the dielectric constants of materials in the microwave range at the nanoscale. The metrological quantification relies strongly on two main pillars: the SMM calibration [32] and the traceability of dimensional measurements. 

Based on the measurements of the PZT and PMN-PT samples, we identify an important interplay between the surface roughness and the thickness of the top electrode gold pads that strongly impacts the dimensional measurements’ uncertainty. Whenever the surface roughness is comparable to the thickness of the gold electrodes, delineation of the electrodes’ structures becomes difficult using AFM topography. This creates a high degree of uncertainty on the determination of the lateral dimensions of the micro-capacitive structures, as it is the case for the PMN-PT sample. To overcome this difficulty, we use the SMM electrical signal by measuring the lateral dimensions of the gold pad electrodes from their electrical signature. Independently of the surface roughness, the SMM signal stems from the formation of a capacitive structure. Unless the radius of the gold pad electrode is not comparable to the radius of the AFM tip apex, the SMM signal on the electrodes is highly predominant. This ensures a clear contrast and well-defined delineation of the gold pads structures, namely the large ones. Small pads, however, suffer from a low contrast on SMM images since their lateral sizes become comparable to the estimated contact area of the metallic AFM tip on the surrounding substrate. This factor adds additional uncertainties on the dimensional measurements on small sized gold pad electrodes. 

Using the ratio of the electrode’s area from the SMM electrical map to that from the calibrated AFM topography allows to account for tip convolution effects in both measurements. Therefore, we demonstrate the applicability of this approach using the PZT sample, based on the good quality of its surface as measured in topography maps. This ratio is then used to determine the topographical areas of the electrodes based on the good electrical contrast in the case of the PMN-PT sample. We postulate that this approach is general, as it could be adapted for any type of considerably rough surfaces. It therefore offers means to exploit the dielectric constants of such rough surface’ materials for which the topography of deposited top electrodes does not allow a direct lateral dimensions’ measurement.

**Figure 11:**
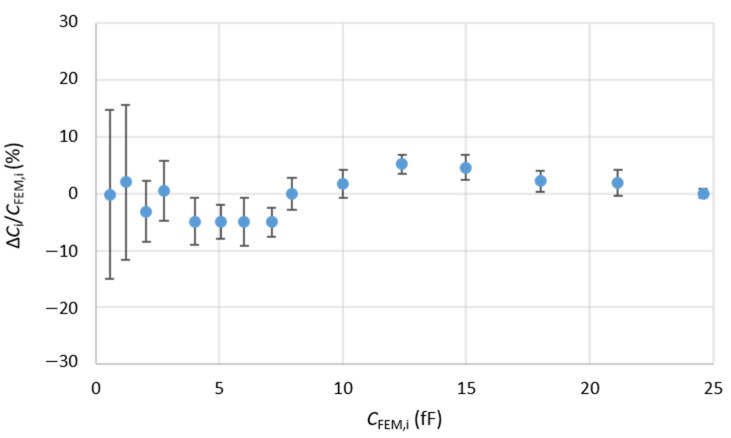
Relative differences Δ*C*_i_/*C*_FEM,i_ (in%) between measured *C*_exp,i_ and FEM calculated *C*_FEM,i_ capacitance values for 15 capacitors Ci of the PZT sample at 3.67 GHz. The SMM has been calibrated using the three capacitors of calculated capacitances 0.56, 7.93, and 24.69 fF.

Nonetheless, the arguments discussed here are based on the essential use of a good quality sample surface such as that of the PZT sample. It naturally follows that the PZT could then be established as a new reference sample with an extended range of capacitance calibration (0.56 to 24.69 fF) compared to the MC2 samples. In a similar fashion to the work reported in [32], we plot the relative error in percent between the FEM-calculated and the measured capacitance of PZT sample, using the PZT itself as the calibration sample of the SMM measurement. Figure 11 shows that the deviations of the measured PZT capacitance values in this case compared to the calculated values do not exceed 6% in absolute value over the full capacitance range. The error bars are calculated from the deviation of the dispersion of 27 capacitance values measured on a given capacitor C_i_, by considering the 3 values *C*_FEM,j_, *C*_FEM,j_ + *u*_CFEM,j_, and *C*_FEM,j_ − *u*_CFEM,j_ for each of the three reference capacitors C_j_ used to calibrate the SMM, where *C*_FEM,j_ is the calculated capacitance of *C*_j_ and *u*_CFEM,j_ the corresponding uncertainty.

This result validates the effective use of the PZT sample as a new reference sample for calibration and measurement of high-κ dielectric constants with an extended range of reference capacitances. It remains interesting to notice that this argument puts forward a validated method for defining new standard samples with a tailored range of capacitances and dielectric constants. The well-characterized MC2 standard samples [32] remain however the starting point of this approach. A clear delineation of the top electrodes topography and SMM electrical signature constitute the key elements for a successful new reference sample candidate. 

## 5. Conclusions

We reported on the metrological quantification of the dielectric constants in the microwave range for two high-κ materials, PZT and PMN-PT. We showed that uncertainties on the lateral dimensions of the gold electrodes in the micro-capacitive structures constitute a large contribution to the combined uncertainty on the measurement of the dielectric constants. We were able to extract the relative permittivity of the PZT sample from SMM imaging (ε_r_ = 445 ± 9). For rough sample such as the PMN-PT, we have demonstrated a new approach to extract dimensions of such structures using their electrical signatures from SMM imaging. This allowed us to measure the dielectric constants ε_r_ = 641 ± 44 for the PMN-PT sample. In relative value, the combined uncertainties were determined at 3.5% and 6.9% for the measurements on PZT and PMN-PT, respectively. The dielectric constant value of these two materials seems to decrease with the frequency in the range of 1.45–5.9 GHz.

Our analysis emphasizes the crucial role that the high-κ surface roughness plays in defining the quality of the interface under the top gold pad electrodes and in introducing considerable effects of parasitic capacitances. We reported on the presence of voids and structural imperfections at the gold/high-κ interface imaged in SEM on cross-section samples. We showed that these interfacial defects lead to size-dependent variations in the micro- and nanocapacitor structures induced by parasitic capacitances.

We finally argued on possible routes to identify new standard calibration samples with extended range of capacitances by following the workflow described in this work and using MC2 standards as a starting calibration step.

## Figures and Tables

**Table 1:** Measured areas *A*_topo_ (PZT) and corresponding uncertainties *u*_Atopo_(PZT) for the 15 gold pads on the PZT sample, the correction factor *N* used to correct area values measured on the PMN-PT sample.

Pad Number	*A*_topo_ (PZT) (µm^2^)	*u*_Atopo_ (PZT) (%)	*N*
1	0.02	7.8	4.47
2	0.05	9.5	2.48
3	0.10	3.1	2.22
4	0.16	3.3	1.99
5	0.24	2.6	1.70
6	0.35	1.5	1.56
7	0.45	1.3	1.47
8	0.56	1.3	1.47
9	0.70	1.7	1.47
10	1.02	1.3	1.34
11	1.40	1.1	1.29
12	1.83	1.2	1.32
13	2.34	1.0	1.28
14	2.92	1.4	1.24
15	3.56	0.5	1.21

**Table 2:** Uncertainty sources included in the current metrological workflow presented in this work and the combined uncertainty *u*c corresponding to the dielectric constant values measured on PZT and PMN-PT samples.

Uncertainty Sources	PZT *u* (%)	PMN-PT (1st Series) *u* (%)	PMN-PT (2nd Series) *u* (%)
Capacitance calculation	(2.5; 9.7)	(6.5; 20.5)	(6.5; 23.6)
Area, *u*_A_	(1.3; 9.5)	(3.5; 19.7)	(3.4; 22.9)
Thickness, *u*_d_	2.1	5.5	5.5
Capacitance measurements	3.2	3.3	3.2
Type-A (Histogram, repeatability)	(0.1; 0.2)	(0.2; 0.6)	(0.2; 0.6)
SMM calibration	3.1	3.1	3.1
Others	<1.8	<1.8	<1.8
Combined uncertainty, *u*_c_ (%)	3.5	9.1	10.6

## Data Availability

The data presented in this study are available on request from the corresponding author.
